# Hybrid convergent ablation versus endocardial catheter ablation for atrial fibrillation: A systematic review and meta‐analysis

**DOI:** 10.1002/joa3.12653

**Published:** 2021-11-02

**Authors:** Mohammed Mhanna, Azizullah Beran, Ahmad Al‐Abdouh, Hazem Ayesh, Omar Sajdeya, Omar Srour, Mahmoud Alsaiqali, Odai H. Alhasanat, Cameron Burmeister, Abdelrhman M. Abumoawad, Paul Chacko

**Affiliations:** ^1^ Department of Internal Medicine University of Toledo Toledo OH USA; ^2^ Department of Internal Medicine Saint Agnes Hospital Baltimore MD USA; ^3^ Department of Internal Medicine State University of New York Downstate Medical Center Brooklyn NY USA; ^4^ Department of Endocrinology University of Toledo Toledo OH USA; ^5^ Department of Internal Medicine University of Missouri Kansas City Kansas City MO USA; ^6^ Department of Cardiovascular Medicine University of Toledo Toledo OH USA

**Keywords:** ablation, atrial fibrillation, endocardial, epicardial, hybrid

## Abstract

**Introduction:**

Endocardial catheter ablation (ECA) for atrial fibrillation (AF) has limited efficacy. Hybrid convergent procedure (HCP) with both epicardial and endocardial ablation is a novel strategy for AF treatment. In this meta‐analysis, we aimed to evaluate the efficacy and safety of HCP in AF ablation.

**Method:**

We performed a comprehensive literature search for studies that evaluated the efficacy and safety of HCP compared with ECA for AF. The primary outcome was freedom of atrial arrhythmia (AA). The secondary outcome was the periprocedural complication rate. Pooled relative risk (RR) and corresponding 95% confidence intervals (CIs) were calculated using the random effects model.

**Results:**

A total of eight studies, including 797 AF patients (mean age: 60.7 ± 9.8 years, 366 patients with HCP vs. 431 patients with ECA alone), were included. HCP showed a higher rate of freedom of AA compared with ECA (RR: 1.48, 95% CI: 1.13–1.94, *p* = .004). However, HCP was associated with higher rates of periprocedural complications (RR: 3.64, 95% CI: 2.06–6.43; *p* = .00001). Moreover, the HCP had a longer procedure time and postprocedural hospital stay.

**Conclusions:**

Although hybrid ablation was associated with a higher success rate, this should be judged for increased periprocedural adverse events and extended hospital stay. Prospective large‐scale randomized trials are needed to validate these results.

## Graphical abstract

Graphical abstract showing the outcomes of our study. Although hybrid ablation was associated with a higher success rate, this should be judged for increased periprocedural adverse events and extended hospital stay.

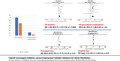

**Abbreviations**
AA: atrial arrhythmia
AADs: antiarrhythmic medications
AF: atrial fibrillation
CI: confidence interval
ECA: endocardial catheter ablation
HCP: hybrid convergent procedure
LA: left atrium
LAPW: left atrial posterior wall
Per‐LSPer AF: persistent or long‐standing persistent AF
PVs: pulmonary veins
RR: relative risk

## 1 INTRODUCTION

Atrial fibrillation (AF) is the most common arrhythmia and continues to be a worldwide health burden.^1^ The prevalence of AF in the United States is rising, with 12.1 million people are expected to have AF by 2030.^1^ Conventional endocardial catheter ablation (ECA) is the mainstay interventional treatment modality of AF and targets mostly pulmonary veins (PVs) isolation.^2^ However, the success rate of ECA is still unsatisfactory, with only half of the patients attaining freedom from atrial tachyarrhythmias at 1‐year follow‐up.^2^ Furthermore, the creation of endocardial transmural lines during the procedure possesses a possible risk of esophageal, lung, and phrenic nerve injury.^3^

Since its advent in the late 1980s, epicardial ablation for AF with various surgical approaches has continued to evolve.^4^ The relative safety varies with the different techniques used, with comparable success rates to the ECA approach.^4^

Hybrid convergent procedure (HCP), which combines both epicardial and endocardial ablation approaches, has emerged to utilize the advantages of both approaches. During the last decade, HCP has gained increasing acceptance in clinical practice, with several reports of durable antiarrhythmic outcomes.^5^ However, studies analyzing the clinical outcomes of HCP are currently limited by small sample sizes. Therefore, we conducted this meta‐analysis to evaluate all the available evidence to better assess the efficacy and safety of the hybrid convergent ablation HCP for atrial fibrillation.

## 2 METHODS

### 2.1 Data sources and search strategy

We performed a comprehensive search for published studies indexed in PubMed/MEDLINE, EMBASE, and the Cochrane Central Register of Controlled Trials from inception to March 30, 2021. We also performed a manual search for additional relevant studies using references of the included articles. The following search terms were used: (“atrial fibrillation” or “AF”), (“hybrid” or “convergent” or “surgical‐transcatheter” or “thoracoscopic‐transcatheter” or “epicardial”), and (“endocardial ablation” or “catheter ablation” or “radiofrequency ablation”). The search was not limited by language, study design, or country of origin. Table S1 describes the full search term used in each database searched.

### 2.2 Study selection

We followed the preferred reporting items for systematic reviews and meta‐analyses (PRISMA) and the meta‐analysis of observational studies in epidemiology (MOOSE) guidelines to screen the studies.^6^, ^7^ We included full texts and abstracts of randomized controlled trials, cohort studies, and case–control studies. We excluded single‐arm studies, animal studies, case reports, case series, reviews, editorials, and letters to editors. Two investigators (MM and AB) independently screened and selected the studies for the final review. Discrepancies were resolved by a third investigator (AA).

### 2.3 Data extraction

We extracted the following data from the final studies: the last name of the first author, publication year, study design, country of origin, follow‐up duration, sample size, efficacy endpoints (the freedom of atrial arrhythmia by the time of the last follow‐up), and safety endpoints (including periprocedural complications such as pericardial effusion, atrio‐esophageal fistula, cerebrovascular accident, and death). Also, we extracted data for the number of patients who underwent HCP or ECA, their age, and baseline comorbidities (including diabetes mellitus, hypertension, body mass index) and preprocedural characteristics (including left ventricular ejection fraction [LVEF], left atrial [LA] diameter, percentage of persistent or long‐standing persistent [Per‐LSPer] AF, pervious treatment with amiodarone, previous ablations, and CHA2DS2‐VASc). Finally, we extracted procedural details, endocardial, and fluoroscopic times as well as the postprocedural hospital stay.

### 2.4 Outcomes

The primary outcome of our meta‐analysis was freedom of atrial arrhythmia (AA) by the time of the last follow‐up. Total AA is defined as a composite of AF, sustained atrial tachycardia (AT), and atypical atrial flutter (AFL) after the index procedure.

Our secondary outcome was the rate of periprocedural complications through 30 days of the index procedure. Complications include the development of stroke, bleeding events that required intervention, pericardial effusion, cardiac tamponade, atrio‐esophageal fistula, infections, phrenic nerve paralysis, and death.

### 2.5 Statistical analysis

The meta‐analysis was performed using Review Manager 5.3 (Cochrane Collaboration, Copenhagen, The Nordic Cochrane Centre). The random effects model was used to calculate the weighted pooled risk ratio (RR) and corresponding 95% confidence intervals (CI). We performed a subgroup analysis for the primary outcome based on the timing of HCP and the use of antiarrhythmic medications. We also performed a subgroup analysis for the safety outcome based on the surgical access used in the HCP. A *p* value of <.05 was considered statistically significant. Heterogeneity was assessed using the Higgins *I* ^2^ index, where *I* ^2^ values >50% implied the presence of substantial heterogeneity.^8^

### 2.6 Quality assessment

We assessed the quality of the included studies using the Newcastle‐Ottawa Scale for observational studies and the Revised Cochrane risk‐of‐bias tool for randomized trials (RoB 2) for RCTs.^9^, ^10^ Two authors (MM and OS) independently assessed each study for bias. Discrepancies were resolved by consensus. We did not evaluate for publication bias in our study because of the limited number of included studies.^11^

## 3 RESULTS

### 3.1 Study selection

**FIGURE 1:**
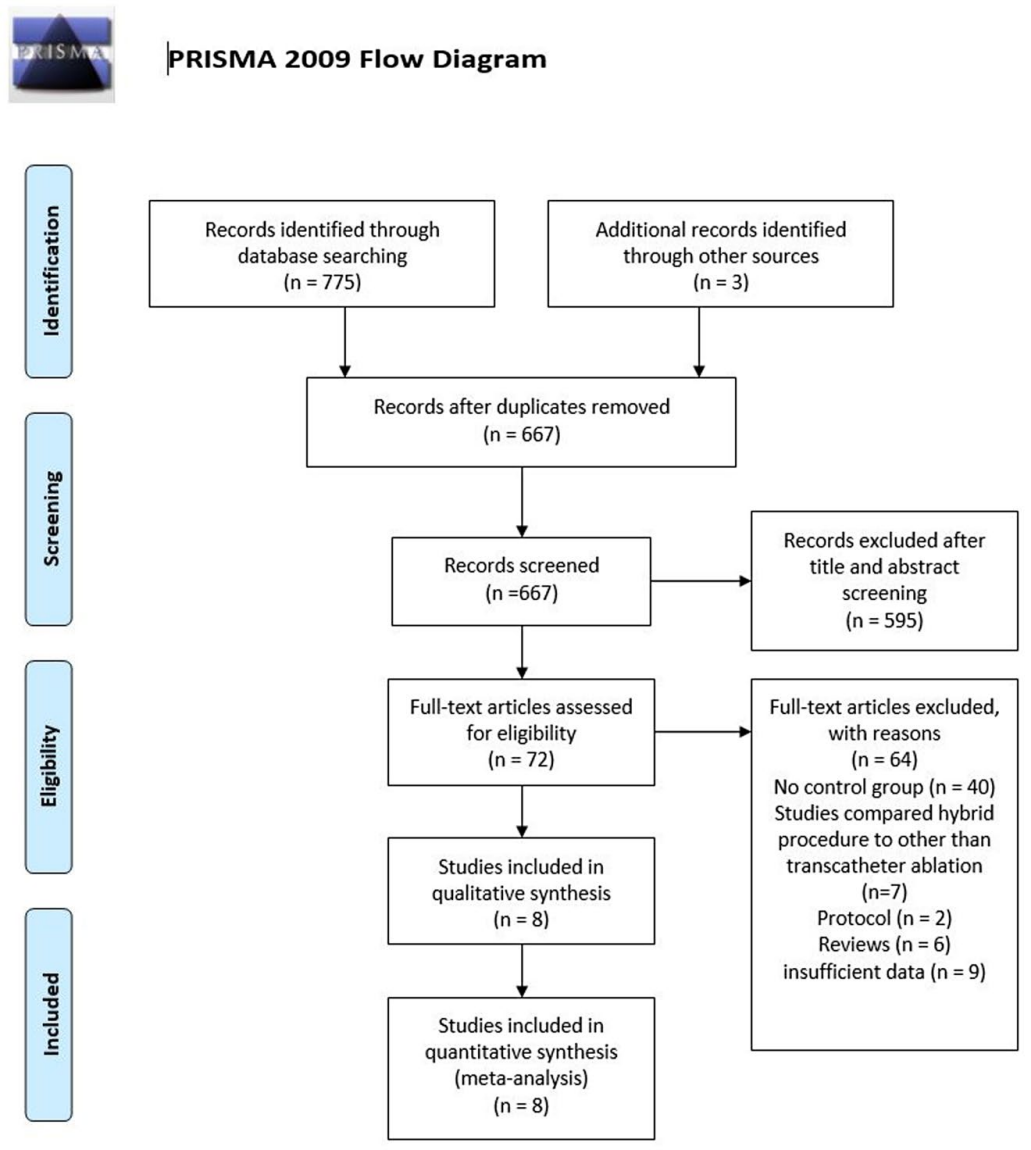
PRISMA flow diagram for the selection of studies

A total of 667 studies were retrieved by our search strategy. Among these, 72 were eligible for the systematic review. Subsequently, we excluded 64 studies that were not relevant, had insufficient data, single‐arm studies, or being a prognostic study. Finally, eight studies met our inclusion criteria and were included in the meta‐analysis.^12^, ^13^, ^14^, ^15^, ^16^, ^17^, ^18^, ^19^ Figure 1 shows the PRISMA flow chart that illustrates how the final studies were selected.

### 3.2 Study characteristics

Table 1 shows the characteristics of the eight studies that were included in our meta‐analysis. The studies included a total of 797 AF patients, of whom 366 underwent HCP and 431 underwent ECA. The studies were published between 2011 and 2020. Based on the country of origin, four studies originated from the United States, one from South Korea, one from Slovenia, one from the United Kingdom, and one multicentric from the United States and the United Kingdom. Based on study design, three studies were randomized controlled trials, four were retrospective cohorts, and one was a prospective cohort. All the included studies were full‐text publications. The mean age was 60.7 ± 9.8 years, and males represented 77.4% of total patients. Around 93% of the entire study population had Per‐LSPer AF, 27% treated previously with amiodarone, and 18.7% underwent prior ablation for AF. Table 2 summarizes the baseline comorbidities and preprocedural characteristics, including LVEF, LA diameter, and CHA2DS2‐VASc score.

**TABLE 1:** Characteristics of studies included in the meta‐analysis

Study	Design	Origin	Follow‐up duration, mean ± SD or mean	Inclusion criteria	AAD use	AA detection
DeLurgio, 2020	RCT	Multicentric, United States, United Kingdom	18	‐ Age 18–80 ‐ Symptomatic persistent AF ‐ Refractory or intolerant to AAD ‐ LA size ≤6.0 cm	Allowed	‐ 24‐h Holter at 6 and 12 months ‐ 7‐day Holter at 18 months
Edgerton, 2016	RCT	United States	24	‐ LSPAF ‐ LAD >4.5 cm ‐ Failed AAD	Allowed	EKG, 7‐day Holter
Genev, 2017	Retrospective Cohort	United States	24	‐ Not received prior invasive AF therapy	Allowed	NR
Hwang, 2018	Retrospective Cohort	South Korea	25	‐ Symptomatic drug‐refractory nonvalvular AF ‐ No prior history of ablation for AF	Allowed	24‐h Holter
Jan, 2018	RCT	Slovenia	30.5 ± 6.9	‐ Paroxysmal AF	Allowed	Implantable loop recorder, monitoring
Kress, 2016	Retrospective Cohort	United States	16	‐ Persistent or LSP AF	Allowed	EKG, loop recorder, Holter
Maclean, 2020	Retrospective Cohort	United Kingdom	30.5 ± 13.3	‐ Persistent AF ‐ No previous cardiac surgery, abdominal surgery, or a contraindication to anticoagulation	Allowed	EKG, 72‐h Holter, Deviinterrogation if pacemaker in place.
Mahapatra, 2011	Prospective cohort	United States	20.7 ± 4.5	‐ Persistent or LSP AF ‐ Failed at least one AAD and one catheter ablation	Allowed	EKG, 7‐day Holter, 24‐h Holter, telephone.

Abbreviations: AA, atrial arrythmia (AF, Atrial flutter, or atrial tachycardia); AAD, antiarrhythmic medications; AF, atrial fibrillation; LA, left atrium; LAD, left atrium diameter; LSP, long standing persistent.

**TABLE 2:** Baseline patient characteristics included in the meta‐analysis

	No of studies	All Patients (*N* = 797)	HCP (*N* = 366)	ECA (*N* = 431)	*p*‐value
Age, year	8	60.7 ± 9.8	61.5 ± 10.1	60 ± 9.5	.03
Male	8	77.4% (617/797)	83.1% (304/366)	72.6% (313/431)	<.01
BMI	5	34.9 ± 12.3	35.2 ± 12.4	34.7 ± 12.3	NS (0.63)
Hypertension	7	62.3% (443/711)	64.1% (207/323)	60.8% (236/388)	NS (0.37)
Diabetes mellitus	6	17% (95/558)	12.7& (28/221)	19.9% (67/337)	.03
CHADS2 score	5	1.8 ± 1.9	1.7 ± 1.5	1.9 ± 2.2	NS (0.22)
LA diameter, cm	6	4.5 ± 0.85	4.5 ± 0.8	4.5 ± 0.9	NS (1.00)
LVEF, %	7	55.5 ± 10.2	55.3 ± 10.2	55.7 ± 10.3	NS (0.6)
Per‐LSPer AF, %	7	92.9% (653/703)	93% (320/344)	92.7% (333/359)	NS (0.89)
Previous amiodarone	3	27.2% (70/257)	25.5% (36/141)	29.3% (34/116)	NS (0.5)
Previous ablation	6	18.7% (129/688)	21.1% (67/318)	16.7% (62/370)	NS (0.15)
Procedure time, min	5	266.1 ± 84.98	304.9 ± 78.8	224.1 ± 70.3	<.0001
Endocardial time, min	5	139.8 ± 83.3	112.6 ± 56.4	169.3 ± 96.8	<.0001
Fluoroscopy time, min	5	34.1 ± 26.9	29.8 ± 25	38.8 ± 28.2	<.0001

Abbreviations: BMI, Body mass index; ECA, Endocardial catheter ablation; HCP, hybrid convergent procedure; LA, Left atrium; LVEF, Left ventricular ejection fraction; Per‐LSPer, Persistent‐ longstanding persistent.

All studies defined AF recurrence as any atrial arrhythmia lasting more than 30 s after the 3‐month blanking period. Follow‐up duration ranged from 16 to 30.5 months.

Genev et al. additionally compared the HCP with another invasive procedure (Complete Cox‐maze),^14^ whereas the rest of the studies compared HCP with ECA. Five studies compared the freedom of AA with or without antiarrhythmic medications (AADs).^12^, ^13^, ^15^, ^16^, ^18^ Five studies reported procedural, endocardial, and fluoroscopy times.^12^, ^13^, ^16^, ^17^, ^19^ Three studies reported the average postoperative hospital stay.^15^, ^17^, ^19^

### 3.3 Procedural characteristics

Five studies performed HCP via transdiaphragmatic subxiphoid incision, two via thoracoscopy, and one via mini‐thoracotomy approach. Three studies performed staged HCP, whereas the rest conducted concomitant endocardial and epicardial HCP. The detailed ablation lesion sets, and sequence of each study were summarized in Table S2.

The assessment of success rates of HCP and ECA was made mainly through Holter monitoring ranging from 24 h to 7 days. Only two studies utilized implantable loop recorder monitoring,^16^, ^17^ and one study interrogated the pacemaker devices when available.^18^

### 3.4 Primary outcomes

**FIGURE 2:**
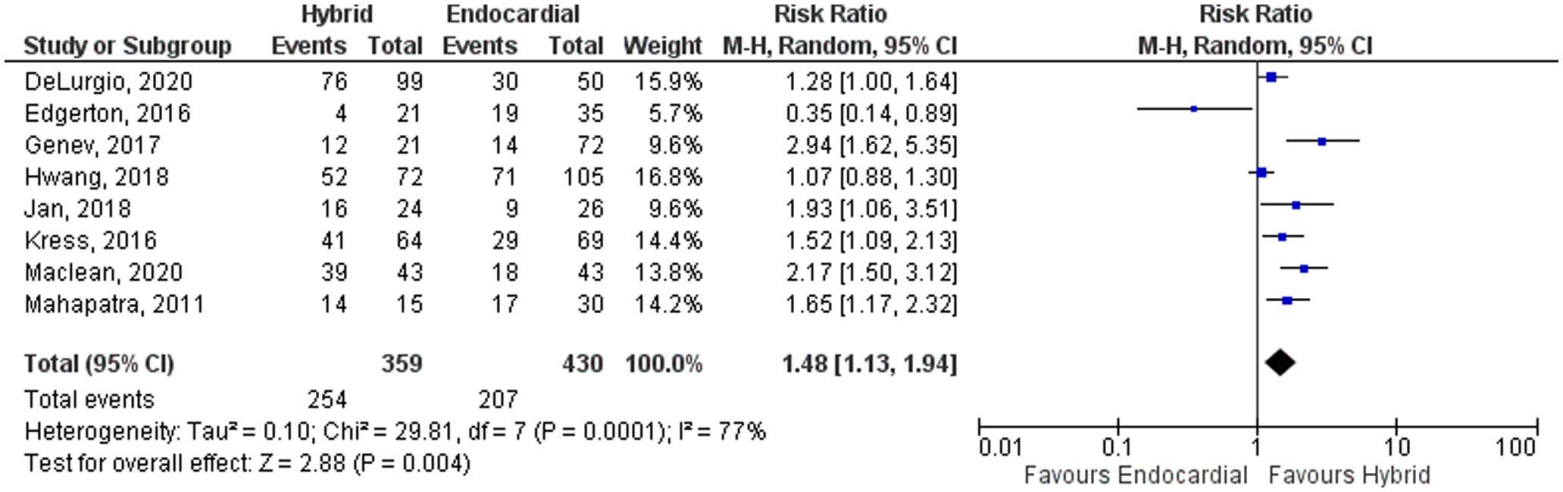
Forest plot comparing hybrid convergent procedure and endocardial catheter ablation regarding the freedom of atrial arrhythmia by the time of the last follow‐up

All the included studies reported the rate of freedom of atrial arrhythmia by the time of the last follow‐up (average of 24 months). HCP showed a higher rate of freedom of AA compared with ECA (RR: 1.48, 95% CI: 1.13–1.94, *p* = .004). However, significant heterogeneity was found (*I* ^2^ = 77%, *p* < .0001) (Figure 2). A sensitivity analysis was conducted by removing one study at a time to reduce heterogeneity and found no significant change in our results (Figure S1). Furthermore, subgroup analysis based on the HCP surgical access failed to improve the observed heterogeneity.

### 3.5 Secondary outcomes

**FIGURE 3:**
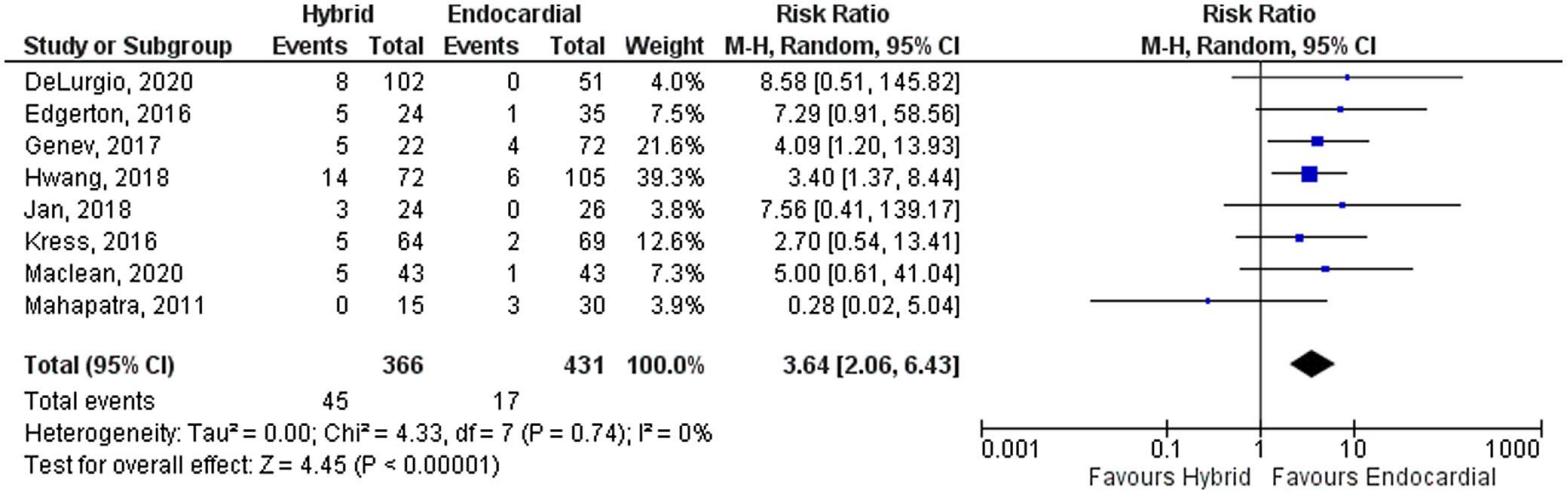
Forest plot comparing hybrid convergent procedure and endocardial catheter ablation regarding the periprocedural adverse events

The rate of periprocedural complications was higher in the HCP group compared with the ECA group (RR: 3.64, 95% CI: 2.06–6.43, *p* = .00001). No significant heterogeneity was found in the measurement of the safety outcome (*I* ^2^ = 0%, *p* = .74) (Figure 3). Forty‐five adverse events were observed in the HCP group compared with only 17 events in the ECA group. Most of the reported complications were procedure‐related bleeding, pericardial effusion, and cardiac tamponade. Furthermore, five deaths were reported in the HCP group. The causes of death included esophageal fistula, large thromboembolic stroke, gastrointestinal bleeding about 2 weeks after the procedure, sudden death at home, and procedure‐related death. No mortality was observed in the ECA group.

### 3.6 Subgroup analyses

**FIGURE 4:**
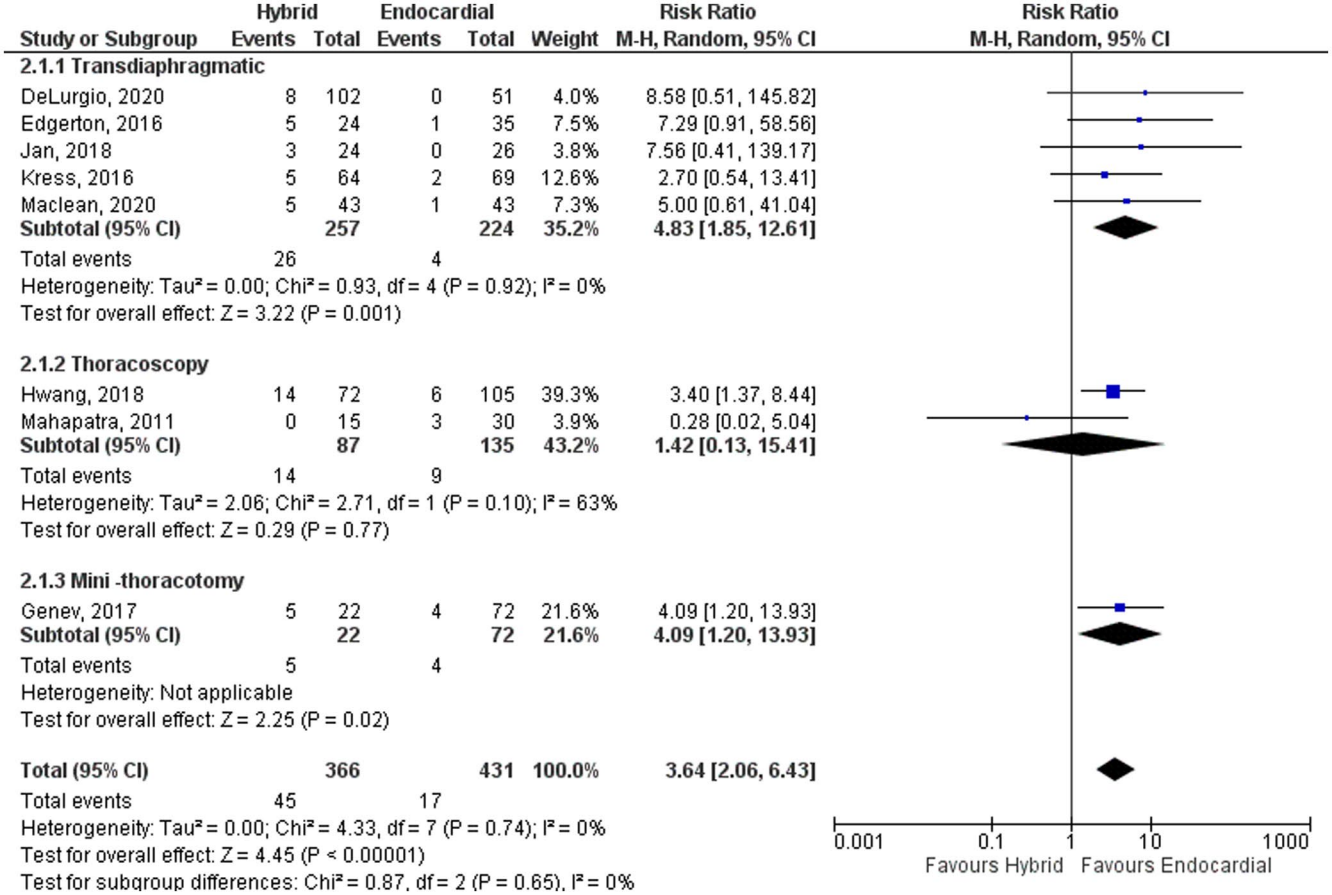
Subgroup analysis comparing hybrid convergent procedure and endocardial catheter ablation regarding the periprocedural complications based on the access of the hybrid convergent procedure

The first subgroup analysis was conducted for the periprocedural complications based on the access of the HCP (transdiaphragmatic vs. thoracoscopy vs. mini‐thoracotomy). Thoracoscopy group of HCP showed better safety profile (RR: 1.42, 95% CI: 0.13–15.41, *p* = .77) (Figure 4). However, the test for subgroup differences was not significant (*I* ^2^ = 0%, chi‐square = 0.87, degrees of freedom = 2, *p*‐value = .65).

The second subgroup analysis was conducted for the freedom of AA based on the timing of the HCP (concomitant vs. staged approaches). HCP and ECA had similar AA freedom rates based on the concomitant (RR: 1.25, 95% CI: 0.83–1.88, *p* = .29) and staged (RR: 1.53, 95% CI: 0.98–2.38, *p* = .06) approaches (Figure S2). The test for subgroup differences was also not significant (*I* ^2^ = 0%, chi‐square = 0.44, degrees of freedom = 1, *p* value = .51).

The third subgroup analysis was conducted for the freedom of AA based on the use of AADs and showed high heterogeneity among both studies with AADs use (*I* ^2^ = 61%, *p*‐value = .04) and studies without AADs use (*I* ^2^ = 53%, *p*‐value = .07). HCP and ECA had similar AA freedom rates based on the AADs use (RR 1.05, 95% CI 0.61–1.80, *p* = .87) and without AADs use (RR 1.47, 95% CI 0.99–2.18, *p* = .06) (Figure S3).

### 3.7 Additional analyses

HCP was associated with significantly prolonged hospital stay compared with the ECA group (mean difference [MD], 3.91 [days], 95% CI: 1.68–6.14; *p* = .0006) (Figure S4).

HCP had longer procedure time than ECA (MD: 89.93 [min], 95% CI: 47.71–131.94, *p* = .0001); however, the endocardial time was shorter in the HCP group (MD: −73.74 [min.], 95% CI: −119.51, −27.96, *p* = .002). No difference was found regarding the fluoroscopy time (MD: −6.07 [min], 95% CI: −23.18, 11.05, *p* = .49). Significant heterogeneity was observed in all reported outcomes (Figure S5).

### 3.8 Quality assessment

We assessed the quality of the included studies by using the Newcastle‐Ottawa Scale for cohort studies and the Revised Cochrane risk‐of‐bias tool for randomized controlled trials, as shown in Tables S3 and S4. All studies scored low to moderate in quality assessment.

## 4 DISCUSSION

This study was a systematic review and meta‐analysis of all studies investigating the efficacy and safety of the hybrid convergent procedure compared to the conventional endocardial catheter ablation for atrial fibrillation. Our meta‐analysis demonstrated that the HCP significantly improved the freedom of atrial tachyarrhythmia. However, it was associated with higher perioperative complications, but HCP through thoracoscopy access showed a better safety profile. Furthermore, HCP was associated with longer procedure time and hospital stay.

Following conventional endocardial ablation, the recurrence rate of AF varies significantly among different studies; early recurrences (within the first 3 months) occur in almost half of the patients after ECA.^20^ Late recurrence (after 3 months) was observed in more than 40% of patients as detected by continuous rhythm monitoring in the CIRCA‐DOSE trial.^2^ Thus, repeated procedures and the use of maintenance antiarrhythmic medications are usually necessary to achieve acceptable success rates.^21^ The rate of repeated ablation procedures may reach up to 80%.^22^ Despite added strategies beyond PV isolation, the success rate did not remarkably improve.^23^

A combined epicardial and endocardial ablation strategy (the hybrid convergent procedure) has been developed to fill the gap of the conventional endocardial catheter ablation. Surgical access (usually through transdiaphragmatic subxiphoid incision, a mini‐thoracotomy, or thoracoscopic approaches) is used to access into the pericardial space to facilitate epicardial ablation, mainly to isolate the posterior wall of the left atrium,^5^ after which endocardial ablation is done by an electrophysiologist to complete the isolation of the pulmonary veins. Furthermore, verifications of the epicardial ablation efficacy can be done during the endocardial portion, and further ablation to isolate the mitral isthmus, cavotricuspid isthmus, or coronary sinus can be done if necessary.^5^ This combined approach facilitates the isolation of both the posterior wall of LA and the PVs; both share the same embryological origin and the arrhythmogenic electrophysiologic potential.^24^

In our study, the rate of late AA recurrence for the ECA group was significantly higher than HCP (51.86% vs. 29.25%, respectively). The enhanced success risk of HCP could be explained by additional ablation of arrhythmogenic targets beyond the isolation of pulmonary veins and robust lesion formation through direct epicardial‐catheter contact.^25^ However, the ECA strategy showed a better safety profile than the HCP. Our results showed that the procedural‐related complications rate was almost three times higher in HCP (12.3% in HCP vs. 3.9 in ECA). Our results are consistent with Khan et al., who reported an overall HCP complication rate of 10%.^26^ Furthermore, the meta‐analysis by Pearman et al. showed no difference between the hybrid procedure and epicardial ablation in the prevention of AA recurrence; moreover, the HCP was associated with higher complication rates (7.3% vs. 2.8%, RR = 2.6).^27^ The safety and efficacy of HCP could be potentially affected by the various approaches to the procedure. The review article by Khoynezhad et al. summarized the outcomes of 15 studies that utilized the HCP in AF ablation^28^ which showed significant variability in the AF freedom outcome (ranging between 19% and 94%) and the safety profile (reported complications rate up to 24%). This significant heterogeneity could be explained by different surgical accesses used, lesion sets applied, the timing of the HCP, type of energy used, and the exclusion of LAA. In our subgroup analysis, thoracoscopic access of HCP showed a better safety profile; however, it was not statistically significant compared with the other two approaches. Furthermore, our results showed that AA freedom rate was similar regardless of whether the HCP was a concomitant or a staged procedure.

Our study showed significant differences in other key outcomes such as the procedure time and the hospital stay. These results are consistent with the meta‐analysis by Zhang et al., which showed a statistically significant difference in these outcomes favoring the ECA strategy.^29^ However, our meta‐analysis included four more studies including two more RCTs, with a larger number of included patients (*n* = 797 patients vs. 331), thus leading to a more robust conclusion on the utility of HCP. Furthermore, we investigated the impact of the procedure access on the rate of periprocedural complications which showed that HCP through thoracoscopy access might have a better safety profile.

For HCP to be successful, it needs a multidisciplinary convergent “team” that integrates skills from cardiothoracic surgery and electrophysiology as well as perioperative coordination because it may require medications adjustment and deal with the potential postoperative complications. In addition, effective patient selection is an important aspect of success. During the HCP, epicardial ablation should be the first component. Under endoscopic observation, a closed‐irrigation, unipolar RF catheter device is typically utilized for epicardial ablation.^30^ To access the left atrial posterior wall (LAPW), the device is placed by a pericardioscopic cannula and moved in the pericardial space with the use of the cannula and endoscope. As the temperature approaches 60°C, the RF energy delivery should achieve 124 coagulation, but not to the point that tissue vaporization occurs. Lesions are typically overlapped across the whole LAPW to improve contiguity and transmurality, minimizing gaps and resulting in a uniform zone of electrical silence. Then, the endocardial component complements the epicardial one by touching up the LAPW lesion set if needed based on an electro‐anatomic map and by performing additional ablation as required based on the individual patient procedure and clinical characteristics.^30^

There are certain limitations to our meta‐analysis. First, the ablation procedures were not standardized among the included studies; however, in most of the study cohort, the posterior wall of the left atrium and the pulmonary veins were ablated. Second, the success rate of ablative procedures was assessed with different approaches. In most of the studies, only symptomatic recurrences proven by EKG or Holter monitor were counted as failures; only two studies employed an implantable loop recorder to confirm the AA recurrence. Third, the included trials were of a single‐blinded design. Therefore, investigator bias cannot be undermined. Last, we could not perform publication bias due to the small number of included studies.

However, there are several strengths to our meta‐analysis. First, to our knowledge, this is the first meta‐analysis to include eight studies with three RCTs to compare the clinical outcomes of adjunctive epicardial strategy with conventional ECA strategy in terms of efficacy and safety. Second, we performed a subgroup analysis for the late atrial arrhythmia recurrence rate based on the timing of the HCP and the use of AADs. In addition, no heterogeneity was found in the measurement of our safety outcome.

In conclusion, our meta‐analysis demonstrated that the hybrid convergent procedure for AF ablation was associated with a higher success rate and reduced the risk of late atrial arrhythmia recurrence. However, this should be judged for increased periprocedural adverse events and extended hospital stay. Prospective large‐scale randomized trials are needed to validate these results.

## CONFLICT OF INTEREST

The authors declared no conflict of interest.

## AUTHOR CONTRIBUTIONS

MM and AB conceived and designed the study and critically revised the manuscript. MM, PC, A. Abumoawad, and A. Al‐abdouh designed the study, collected, analyzed, and interpreted the data and drafted the manuscript. HA, MA, CB, OH, O. Sajdeya, and O. Srour collected the data and reviewed the literature. All authors read and approved the final manuscript.

## IRB APPROVAL

This study was deemed exempt by the Institutional Review Board of the University of Toledo, as it was a meta‐analysis of published studies that included de‐identified patient information.

## Supporting information

Supplementary Material
