# Supplementary material for: Hybrid convergent ablation versus endocardial catheter ablation for atrial fibrillation: A systematic review and meta‐analysis
Source: J Arrhythm. 2021 Nov 2;37(6):1459–67. doi: 10.1002/joa3.12653 (PMC8637076; doi:10.1002/joa3.12653)
Supplement: Supplementary file 1 — Supplementary Material [file JOA3-37-1459-s001.docx]

**SUPPLEMENTARY APPENDIX**

**Hybrid Convergent Ablation versus Endocardial Catheter Ablation for Atrial Fibrillation: A Systematic Review and Meta-Analysis**

| Title | Page |
| --- | --- |
| Supplementary Table 1. Search strategy used in each database searched | 2 |
| Supplementary Table 2. Procedural characteristics of the included studies. | 3-4 |
| Supplementary Table 3. The Revised Cochrane risk-of-bias tool for randomized trials (RoB 2) for RCTs | 5 |
| Supplementary Table 4. The Newcastle-Ottawa Scale (NOS) for assessing the quality of nonrandomized studies in meta-analysis | 6 |
| Supplementary Figure 1. Sensitivity analysis for the freedom of atrial arrhythmia. | 7 |
| Supplementary Figure 2. Subgroup analysis comparing hybrid convergent procedure and endocardial catheter ablation regarding the freedom of atrial arrhythmia by the time of last follow-up based on timing of the hybrid convergent procedure. | 8 |
| Supplementary Figure 3: Subgroup analysis comparing hybrid convergent procedure and endocardial catheter ablation regarding the freedom of atrial arrhythmia by the time of last follow-up based on the use of antiarrhythmic drugs: A) on antiarrhythmic drugs, B) off antiarrhythmic drugs. | 9 |
| Supplementary Figure 4: Forest plot comparing hybrid convergent procedure and endocardial catheter ablation regarding the hospital stay. | 10 |
| Supplementary Figure 5: Forest plots comparing hybrid convergent procedure and endocardial catheter ablation regarding: a) procedure time, b) endocardial time, and c) fluoroscopy time | 11 |

Supplementary Table 1: Search strategy used in each database searched.

| Database | Search Strategy | Articles retrieved |
| --- | --- | --- |
| MEDLINE | ("atrial fibrillation"[MeSH Terms] OR ("atrial"[All Fields] AND "fibrillation"[All Fields]) OR "atrial fibrillation"[All Fields] OR "atrial fibrillation"[Text Word]) AND ("hybrid"[Text Word] OR "epicardial-endocardial"[Text Word] OR "surgical-transcatheter"[Text Word] OR "thoracoscopic-transcatheter"[Text Word]) | 411 |
| Embase | ('atrial fibrillation'/exp OR 'atrial fibrillation') AND ('hybrid ablation'/exp OR 'hybrid ablation' OR 'endocardial and epicardial' OR 'surgical-transcatheter' OR 'thoracoscopic-transcatheter') | 289 |
| Cochrane CENTRAL | "atrial fibrillation" AND (hybrid OR 'endocardial and epicardial' OR 'surgical-transcatheter' OR 'thoracoscopic-transcatheter') | 75 |

Supplementary Table 2: Procedural characteristics of the included studies.

| Study | Access | HP, epicardial ablation | HP, endocardial ablation | ECA procedure | LAA exclusion | Conduction block checked | Timing |
| --- | --- | --- | --- | --- | --- | --- | --- |
| DeLurgio, 2020 | TD, Sub-X | Ablation around PV antrum and  across the posterior wall of LA | Isolation of the PVs, and CTI | RFA of the PVs, roof line, CTI line. Optional CFAE | No | Yes | Concomitant |
| Edgerton, 2016 | TD, Sub-X | RFA,  PVs, posterior wall, LOM  (without dissection) and the lateral RA isolation. | RFA. Verification of the surgical  lesions  Further ablation: CS and LAA. If  CFAEs were detected in the LA,  they were ablated | RFA. Isolation of PVs, posterior wall,  LOM, CS, LAA, and RA | No | Yes | Concomitant |
| Genev, 2017 | Mini thoracotomy | NR | NR | NR | NR | NR | NR |
| Hwang, 2018 | Thoracoscopy | Isolation of PVs. Ablation of PV carina, Roof line, Inferior line and ganglionated plexus. Division of LOM. Ablation of SVC -IVC, CTI, and MI line. | PV isolation. Ablation of  CTI, MI, PV carina, Roof line | PV isolation. Ablation of  CTI, MI, PV carina, Roof line | Yes | Yes | Staged |
| Jan, 2018 | TD, Sub-X | PV isolation. Ablation across the posterior LA wall | PV isolation | PV isolation | No | Yes | Concomitant |
| Kress, 2016 | TD | Cryo or RFA,  Isolation of PVs, posterior LA wall | Isolation of PVs with catheter ablation  or the cryoballoon, CFAEs and  linear lesions with RFA | Isolation of PVs with RFA or  cryoballoon, CFAEs and linear  lesions with RFA | No | Yes | Concomitant |
| Maclean, 2020 | TD, Sub-X | Coagulation of the posterior wall of  the LA. DCCV was performed to achieve sinus rhythm  if required. | Isolation of PV, posterior wall. Additional CFEA ablation. DCCV if not in sinus rhythm | Isolation of PV, posterior wall. Additional CFEA ablation. DCCV if not in sinus rhythm | No | Yes | Staged |
| Mahapatra, 2011 | Thoracoscopy | PV ablation. Isolation of SVC, placement of roof line, mitral line,  elimination of ganglia response, LOM  ablation | RFA. If AFL was induced, it  was mapped and ablated. If AF  was induced, checking PVs  isolation, roof line, then MI line | RFA. Antral ablation, roof line, and  CTI line. Ablation of Mitral line,  CS,  SVC  and CFAEs (optional). | Yes | Yes | Staged |

Abbreviations: AF: Atrial fibrillation, AFL: Atrial flutter, CA: Coronary sinus, CFAEs: Complex fractionated atrial electrograms, CTI: Cavo-tricuspid isthmus, DCCV: Direct current cardioversion, ECA: endocardial catheter ablation, HP: Hybrid procedure, IVC: Inferior vena cava, LA: Left atrium, LAA: Left atrial appendage, LOM: Ligament of Marshall, MI: Mitral isthmus, PV: Pulmonary vein, RA: Right Atrium, RFA: Radiofrequency ablation, SA: Surgical ablation, Sub-X: Subxiphoid, SVC: Superior vena cava, TD: Transdiaphragmatic.

Supplementary Table 3: The Revised Cochrane risk-of-bias tool for randomized trials for randomized controlled trials in the meta-analysis.

| Author, year | Bias arising from the randomization process | Bias due to deviations from intended interventions | Bias due to missing data | Bias in measurement of outcomes | Bias in selection of the reported result | low/moderate/serious / critical |
| --- | --- | --- | --- | --- | --- | --- |
| DeLurgio, 2020 | Low | Low | Low | Low | Low | Low |
| Edgerton, 2016 | Moderate | Moderate | Low | Moderate | Low | Moderate |
| Jan, 2018 | Low | Moderate | Low | Low | Low | Low |

Supplementary Table 4: The Newcastle-Ottawa Scale for assessing the quality of nonrandomized studies in the meta-analysis.

|  | Selection | | | | | Outcome | | | |
| --- | --- | --- | --- | --- | --- | --- | --- | --- | --- |
| Study | Representative nest of the exposed cohort | Selection of the non-exposed cohort | Ascertainment of exposure | Outcome not present at baseline | Comparability of the cohort | Assessment of outcome | Enough follow up duration | Adequate follow-up | Total score |
| **Genev, 2017** | * | * | * | NA | * | NA | * | NA | 5 |
| **Hwang, 2018** | * | * | * | NA | * | NA | * | * | 6 |
| **Kress, 2016** | * | * | * | NA | * | * | * | * | 7 |
| **Maclean, 2020** | * | * | * | NA | * | * | * | * | 7 |
| **Mahapatra, 2011** | * | * | * | NA | * | * | * | NA | 6 |

Each asterisk represents one star in the Newcastle-Ottawa Scaling System (NOS). The maximum stars are 2 for comparability and 1 are for all other categories. Each star counts towards the total score. Score of 5 to 6 considered as moderate quality and 7 to 9 as high quality.

Abbreviation: NA: not available.


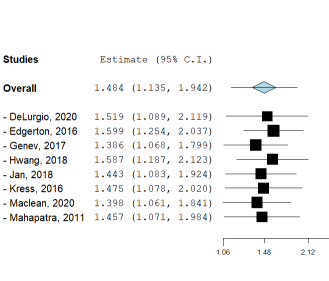


**Supplementary Figure 1:** Sensitivity analysis for the freedom of atrial arrhythmia.


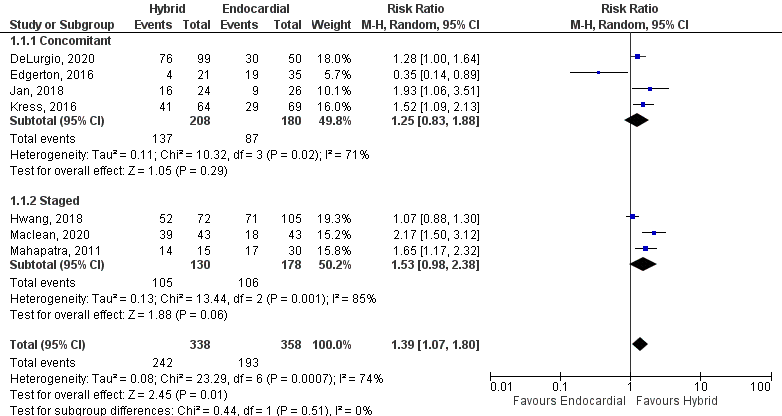


**Supplementary Figure 2:** Subgroup analysis comparing hybrid convergent procedure and endocardial catheter ablation regarding the freedom of atrial arrhythmia by the time of last follow-up based on timing of the hybrid convergent procedure.


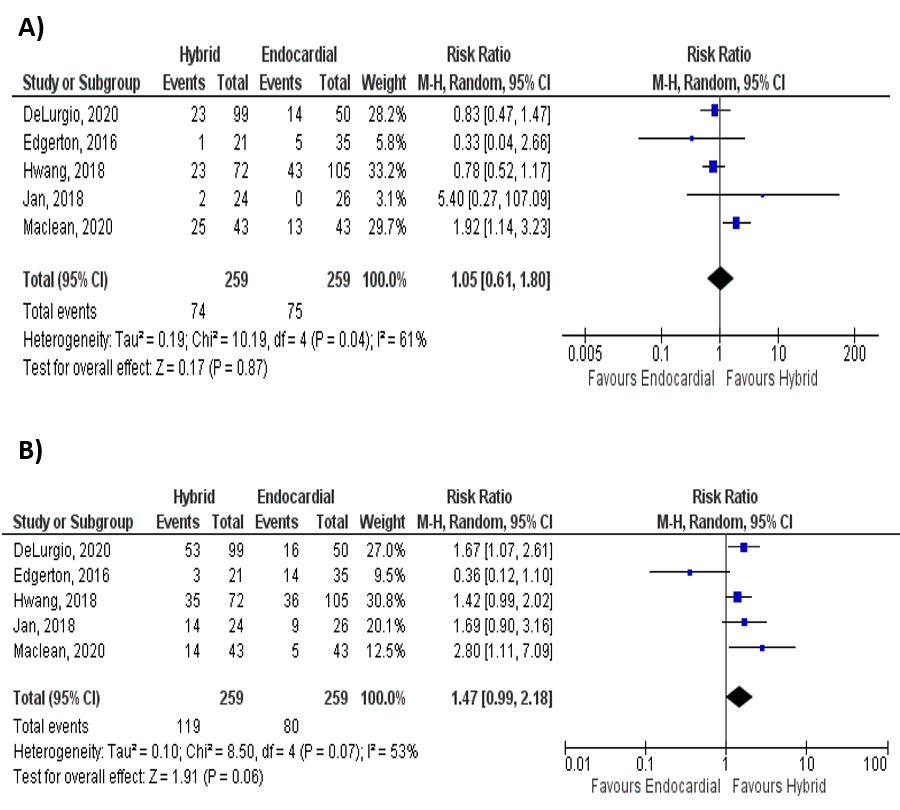


**Supplementary Figure 3:** Subgroup analysis comparing hybrid convergent procedure and endocardial catheter ablation regarding the freedom of atrial arrhythmia by the time of last follow-up based on the use of antiarrhythmic drugs: A) on antiarrhythmic drugs, B) off antiarrhythmic drugs.


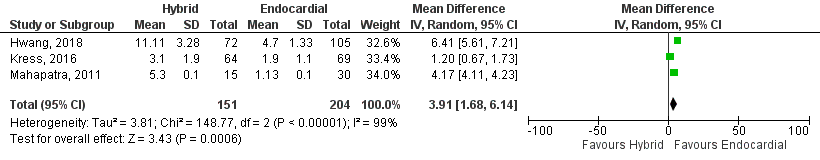


**Supplementary Figure 4:** Forest plot comparing hybrid convergent procedure and endocardial catheter ablation regarding the hospital stay.


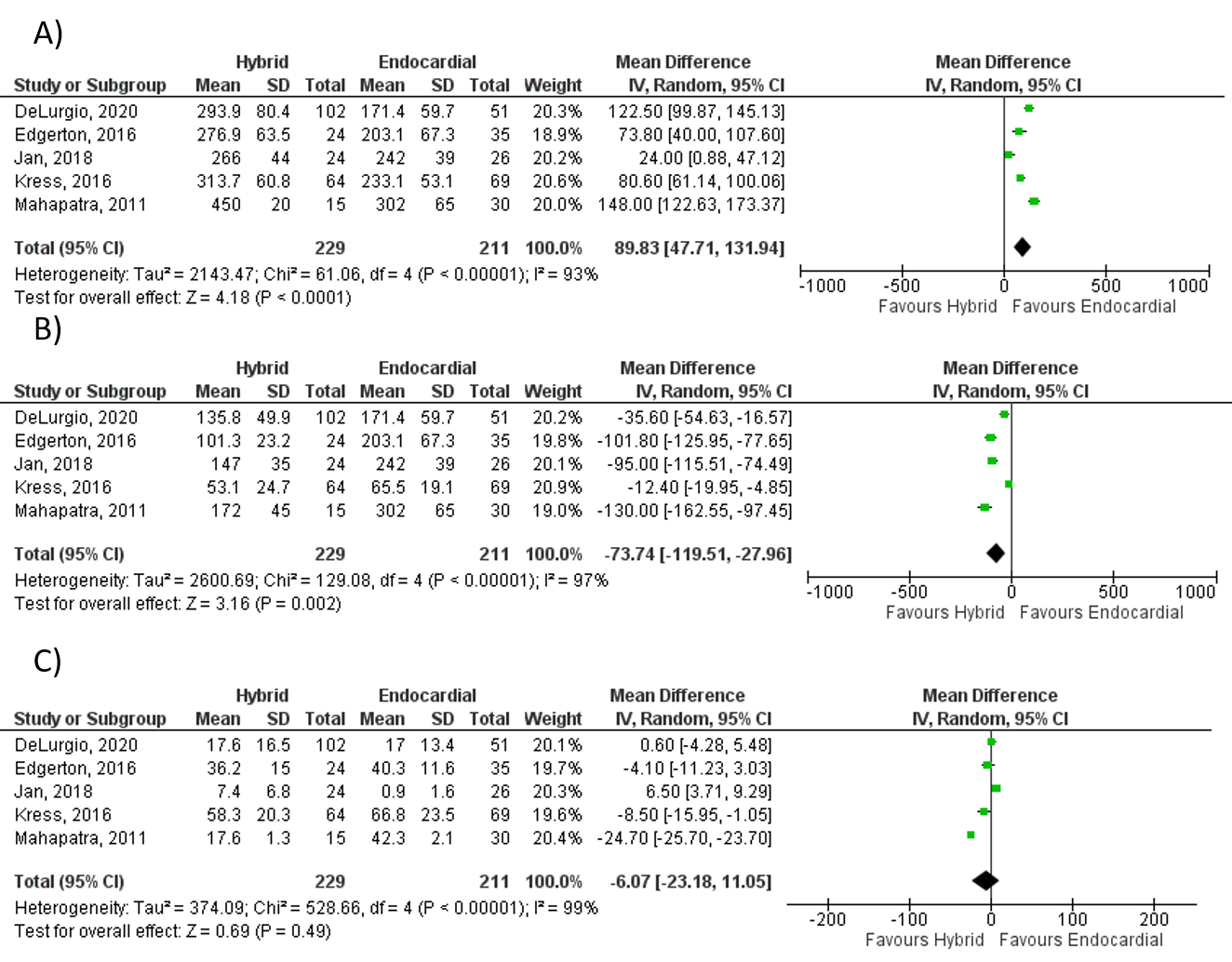


**Supplementary Figure 5:** Forest plots comparing hybrid convergent procedure and endocardial catheter ablation regarding: a) procedure time, b) endocardial time, and c) fluoroscopy time.
